# 1396. Performance of a Telemedicine Travel Clinic

**DOI:** 10.1093/ofid/ofad500.1233

**Published:** 2023-11-27

**Authors:** Adam Pomeranz, Iris Zohar, Gabriele Caliari, Nili Orbach, Yulia Mizrachi, Hadar Mimon, Debby Ben-David, Yasmin Maor

**Affiliations:** Wolfson Medical Center, Tel Aviv, HaMerkaz, Israel; Wolfson Medical Center, Tel Aviv, HaMerkaz, Israel; Wolfson Medical Center, Tel Aviv, HaMerkaz, Israel; Wolfson Medical Center, Tel Aviv, HaMerkaz, Israel; Wolfson Medical Center, Tel Aviv, HaMerkaz, Israel; Wolfson Medical Center, Tel Aviv, HaMerkaz, Israel; Wolfson Medical Center, Tel Aviv, HaMerkaz, Israel; Wolfson Medical Center, Tel Aviv, HaMerkaz, Israel

## Abstract

**Background:**

International travel has become easier and more accessible, with increasing numbers of people embarking trips to diverse destinations worldwide. This trend is accompanied by elderly individuals and families venturing to remote locations, as well as digital nomads. As a result, there is a growing demand for pre-travel medical consultations, which current medical resources in Israel are not able to meet. To address this, we launched a telemedicine-based travel clinic that aims to improve accessibility for Israeli residents.

**Methods:**

The digital clinic is located within a 700-bed medical center in central Israel and offers telemedicine consultations scheduled online. After the appointment, customers receive recommendations and prescriptions via email. They can then make an appointment to receive vaccinations at our on-site in person clinic or at other travel clinics in Israel. From February 1st to March 31st, 2023, we analyzed all cases that receive a digital consultation. Data included demographics, travel destinations, vaccination recommendations, and attendance to the in-person vaccination clinic.

**Results:**

239 customers received a telemedicine consultation. Mean age was 33±17 years, 122 (51.0%) were females, 40 (16.7%) were minors. 18.4%44 () resided outside the pooling area of the hospital. Of these, 81.8%36 () lived in central Israel but had closer clinics to their place of residency requiring personal attendance, 18.2%8 () lived in the North or South of Israel (figure 1). Travel destinations are presented in figure 2. 219 (91.6%) customers received a recommendation for at least one vaccine. 53.0%116 () attended or scheduled an appointment to the vaccine clinic. 103 (47.0%) did not schedule an appointment despite having a recommendation to receive vaccines.

**Figure:**
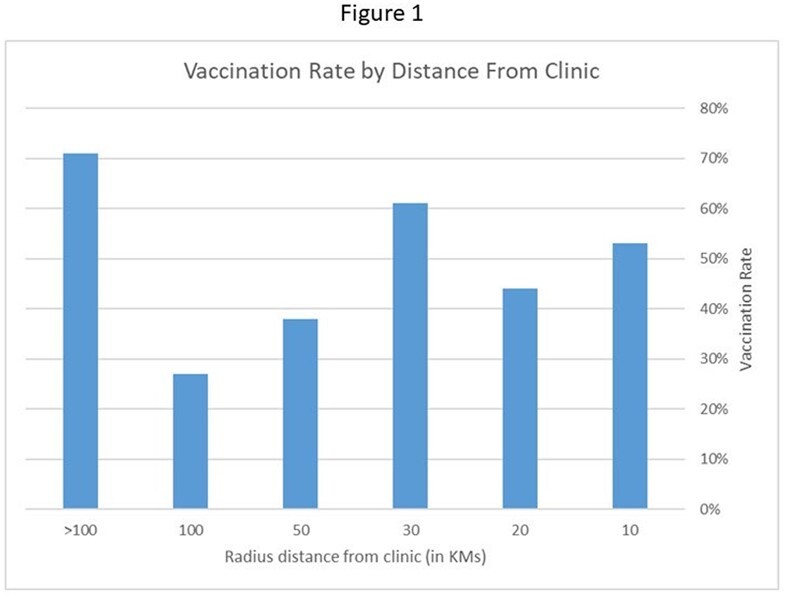

**Figure:**
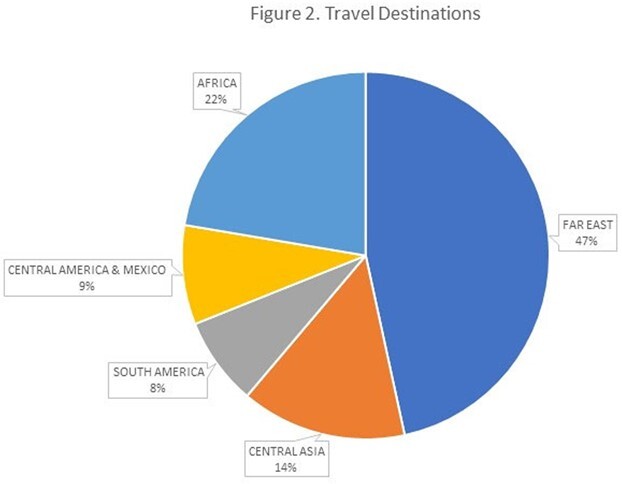

**Conclusion:**

About 50% of patients did not attend or schedule an appointment to the in-person clinic to receive the recommended vaccines. Personal attendance decreased as the residence distance from the hospital increased ( >30 Km), suggesting that customers found closer clinics for vaccination. The telemedicine-based travel clinic simplified pre-travel medical consultations and for 10% of clients who did not need to get vaccines enabled them not to attend the hospital in person.

**Disclosures:**

**Yasmin Maor, MD**, Astra Zeneca: Advisor/Consultant|Astra Zeneca: Honoraria|Gilead: Honoraria|GSK: Honoraria|kamada: Honoraria|Medison: Honoraria|MSD: Advisor/Consultant|MSD: Honoraria|Pfizer: Grant/Research Support|Pfizer: Honoraria

